# Effects of 1-Deoxynojirimycin-Enriched Mulberry Extract on Liver and Gut Health in Broilers Under a High-Fat Diet

**DOI:** 10.3390/ani16182872

**Published:** 2026-09-12

**Authors:** Liqin Zhao, Mingzhu Wang, Yuan Feng, Tao Li, Cangning Zhang, Liang Qu, Dan Shao, Chengmin Li, Manman Shen, Weiguo Zhao

**Affiliations:** 1 Jiangsu Key Laboratory of Sericultural and Animal Biotechnology, School of Biotechnology, Jiangsu University of Science and Technology, Zhenjiang 212100, China; 18717651933@163.com (L.Z.); wangmingzhu129@163.com (M.W.); fengyuan6181@126.com (Y.F.); taoli912@126.com (T.L.); zhangcangning1999@163.com (C.Z.); chengminli@just.edu.cn (C.L.); 2 Key Laboratory of Silkworm and Mulberry Genetic Improvement, Ministry of Agriculture and Rural Affairs, Sericultural Scientific Research Center, Chinese Academy of Agricultural Sciences, Zhenjiang 212100, China; 3 Jiangsu Institute of Poultry Science, Yangzhou 225125, China; liangquyz@126.com (L.Q.); yzshaodan@163.com (D.S.)

**Keywords:** 1-deoxynojirimycin, mulberry, oxidative stress, liver, gut microbiota

## Abstract

In broiler farming, high-fat (HF) diets are frequently incorporated, often leading to hepatic steatosis, oxidative stress, and gut damage. 1-Deoxynojirimycin (DNJ) has emerged as a promising feed additive in animal husbandry due to its significant role in enhancing animal health. This study assessed the protective effects of DNJ-enriched mulberry extract (DNJ) on liver and gut health in broilers subjected to an HF diet, with particular emphasis on the liver-gut interaction. A total of 360 one-day-old Arbor Acres broilers were initially raised on a basal diet for 20 days, after which they were assigned to one of three dietary treatments from days 21 to 42: a control diet (CON), an HF diet, and an HF diet supplemented with 80 mg/kg DNJ (HF + DNJ). By day 42, broilers on the HF diet exhibited increased body weight, feed intake, and body weight gain, alongside reduced feed conversion efficiency. In contrast, DNJ supplementation reduced body weight without affecting feed intake in the HF diet group. It lowered liver T-CHO and LDL-C levels, increased SOD and CAT activities, and decreased MDA. Histological analyses showed that DNJ preserved intestinal mucosal structure and epithelial tight junctions. DNJ supplementation enriched microbes like Actinobacteriota and Baceroidota and reversed the HF-induced Firmicutes-to-Bacteroidota ratio. Furthermore, *Bifidobacterium pullorum* and Bacteroidota were positively correlated with antioxidant markers. In conclusion, dietary DNJ-enriched mulberry extract serves as an effective functional feed substance that mitigates HF-induced metabolic syndromes in broilers by balancing lipid metabolism, enhancing antioxidant defenses, safeguarding intestinal barrier integrity, and restoring healthy microbial community structures along the liver-gut axis.

## Simple Summary

High-fat (HF) diets are widely applied in poultry production to promote growth rates. However, excessive fat intake can disturb lipid metabolism, induce oxidative stress, and impair intestinal function. In this study, we evaluated the effects of DNJ-enriched mulberry extract on liver and intestinal health in broilers challenged with a high-fat (HF) diet. Supplementation with DNJ alleviated hepatic lipid disorder and improved antioxidant status. Meanwhile, DNJ supplementation preserved intestinal morphology and barrier integrity, accompanied by beneficial changes in gut microbial composition. These findings suggest that DNJ-enriched mulberry extract may serve as a potential nutritional strategy to support liver–gut health and mitigate the adverse effects of high-fat diets in broilers.

## 1. Introduction

The application of breeding technology in poultry has significantly increased the growth rate of broilers. Meanwhile, advancements in nutritional regulation and management systems have further enhanced this process. Increasing the metabolizable energy (ME) improves the efficiency of feed conversion to body weight (BW) gain in broilers [1]. However, a high-fat diet often contributes to oxidative stress, fatty liver disease, imbalances in gut microbiota, and cellular damage in poultry, which contributes to the development and progression of steatohepatitis from steatosis [2]. Oxidative stress disrupts the balance of the intestinal environment and alters intestinal permeability, causing damage and triggering a range of intestinal diseases [3]. To combat oxidative stress, restricted feeding regimes are employed; however, these often impair production performance and reduce economic profitability [4]. The use of herbal medicines with antioxidant properties has proven effective in mitigating oxidative damage in chickens fed a high-fat (HF) diet [5]. Yang et al. [6] found that dietary supplementation with perillartine ameliorates lipid metabolism disorders induced by a HF diet in broiler chickens. Sevillano et al. [7] found that supplementation with olive pomace attenuated the enzymatic antioxidant response in broilers fed with soybean oil or peroxidized soybean oil. These studies demonstrate that dietary supplementation with bioactive components plays a protective role in liver and gut health. Therefore, the exploration of high-efficiency, low-cost feed additives is crucial for improving poultry farming.

1-Deoxynojirimycin (DNJ), a polyhydroxy alkaloid primarily extracted from mulberry (*Morus alba* L.), is a potent and well-characterized α-glucosidase inhibitor widely recognized for its ability to suppress postprandial hyperglycemia in humans [8]. Beyond its classic hypoglycemic activity, DNJ exhibits diverse pharmacological properties, including antioxidant, anti-inflammatory, antiviral, anti-obesity, and antitumor effects [9]. Regarding lipid regulation, DNJ has been reported to modulate the gut microbiota and alleviate HF diet-induced non-alcoholic steatohepatitis (NASH) in mice [10], suggesting a protective role mediated via the gut-liver axis [11]. Recently, the application of DNJ in livestock and poultry nutrition has attracted significant interest. In vitro studies indicate that DNJ can alleviate copper-induced oxidative stress in porcine granulosa cells [12] and suppress porcine epidemic diarrhea virus replication by reducing reactive oxygen species (ROS) accumulation [13]. Furthermore, previous work in our laboratory demonstrated that dietary supplementation with DNJ-enriched mulberry extract improves antioxidant capacity and enhances intestinal function in laying hens and broilers; the appropriate supplementation levels were approximately 80 mg/kg of the basal diet or even lower [14,15]. These results indicate that DNJ exerts protective effects when the organism is challenged by oxidative stress. However, excessive doses may exert detrimental effects on the intestinal architecture of geese [16].

Despite growing evidence of the regulatory effects of DNJ on anti-oxidative stress, the integrated mechanisms by which DNJ modulates crosstalk along the liver-gut axis under HF diet-induced metabolic stress remain poorly understood. Given its antioxidant properties, the application of DNJ to combat nutritional and metabolic disorders in modern poultry production holds significant practical and scientific value. Therefore, this study was designed to evaluate the effects of dietary DNJ-enriched mulberry extract on hepatic lipid metabolism, intestinal mucosal morphology, and cecal microbiota composition in broilers fed an HF diet. By utilizing an HF diet-challenged model, this research aimed to validate the efficacy of DNJ supplementation in restoring hepatic lipid metabolism and intestinal barrier integrity, and modulating the liver-gut axis, thereby reinforcing its critical role as a functional feed additive to mitigate metabolic stress in poultry.

## 2. Materials and Methods

### 2.1. Animal Ethics Statement

The present study was approved by the Animal Care and Use Committee of Jiangsu University of Science and Technology (No. GSB202131002).

### 2.2. Experimental Diets and Feeding Trial

A total of 360 one-day-old Arbor Acres (AA) broilers with similar body weight (40.3 ± 1.1 g) were provided by Jiangsu Jinghai Poultry Lo.ltd. (Nantong, China). On day 0, chicks were randomly allocated to identical floor pens in an environmentally controlled facility at Jiangsu University of Science and Technology. Each pen was equipped with a feeder and two nipple drinkers, allowing ad libitum access to commercial broiler feed and water. The lighting program and temperature conditions strictly followed the AA broiler breed guidelines [17]. The environmental temperature was initially maintained at 32 °C for the first 3 days and then gradually reduced by 2–3 °C each week until it reached a constant 20 °C, which was maintained until the end of the trial. On day 21, the broilers were reassigned to pens to ensure a uniform average initial body weight (824.33 g) across all replicates. The birds were randomly allocated to 3 dietary treatments with 8 replicates per treatment and 15 birds per pen. The experimental treatments consisted of a basal diet (CON), an HF diet containing 3240 kcal/kg metabolizable energy (ME), and an HF diet supplemented with 80 mg/kg mulberry leaf extract 1-deoxynojirimycin (HF + DNJ), the detailed ingredient composition and nutrient levels are shown in Table 1. The HF diet was designed according to production practice and previous studies [18,19,20]. DNJ extract (purity ~50%) utilized in this experiment was provided by Xi’an Shengqing Co., Ltd. (Xi’an, China). Briefly, the DNJ-enriched mulberry extract was obtained using a two-stage LSI-D113 cation-exchange resin tandem process. Our laboratory verified the DNJ content to be 49.7% using HPLC, as shown in Figure S1. Simultaneously, we roughly measured the total alkaloid content in the extract at 63.4% using the acid dye colorimetric method, while the contents of polysaccharides (22.1%) and total polyphenols (1.3%) were determined using anthrone colorimetry and the Folin–Ciocalteu colorimetric assay, respectively. The DNJ supplementation levels were designed according to our previous studies [14,15] and the recommended supplementation levels in mice [21,22].

### 2.3. Growth Performance

Individual body weight (BW) and feed consumption per pen were recorded on days 21 and 42 of the trial. Mortality was monitored and recorded daily. These data were used to calculate average daily feed intake (ADFI), average daily body weight gain (BWG), and the feed conversion ratio (FCR) for the corresponding experimental periods.

### 2.4. Sample Collection

On day 42, following a 12-h fasting period, two birds per replicate were randomly selected and humanely euthanized by cervical dislocation for tissue sampling. After opening the abdominal cavity, the liver and intact intestinal tract were quickly excised. The liver was weighed, and its relative weight was calculated and expressed as grams per kilogram (g/kg) of live body weight.

For gut histological analysis, approximately 2-cm segments from the midpoints of the duodenum, jejunum, and ileum were collected and fixed in 4% paraformaldehyde. Concurrently, separate midpoint sections of the jejunum and duodenum were harvested and immediately fixed in glutaraldehyde for transmission electron microscopy (TEM) analysis and scanning electron microscopy (SEM) analysis, respectively. Additionally, cecal contents were collected under sterile conditions, immediately frozen in liquid nitrogen, and stored at −80 °C for 16S rRNA gene sequencing.

### 2.5. Liver and Intestinal Morphology Observations

The fixed liver and intestine tissues were dehydrated, embedded in paraffin, and sectioned at a thickness of 5 μm. The sections were stained with hematoxylin and eosin (H&E) according to standard histological protocols. Morphological observations were performed using a microscope (IX73, Olympus, Tokyo, Japan). For intestinal histomorphometry, villus height and crypt depth were measured in each sample by analyzing at least 10 well-oriented villi per section using a computer-assisted morphometric system. The villus-to-crypt ratio (V/C) was calculated as the ratio of villus height to crypt depth.

### 2.6. Biochemical Levels Assessment

Liver tissue samples were homogenized for biochemical parameter detection. Parameters of aspartate transaminase (AST) and alanine aminotransferase (ALT) were quantified using commercial ELISA kits (Jiancheng, Nanjing, China) following the manufacturer’s protocols. Oxidative stress markers, including malondialdehyde (MDA), superoxide dismutase (SOD), and catalase (CAT), were measured using corresponding kits from Jiancheng. Lipid metabolic parameters, including total cholesterol (T-CHO), triglycerides (TG), low-density lipoprotein cholesterol (LDL-C), and high-density lipoprotein cholesterol (HDL-C), were also assessed with kits from Jiancheng. All operations were strictly carried out according to the manufacturer’s instructions for the respective kit.

### 2.7. Transmission Electron Microscopy

Jejunal tissue samples were sectioned with a scalpel and subsequently post-fixed in a mixture of 1% osmium tetroxide, 0.8% potassium ferrocyanide, and 10 mM calcium chloride, prepared in 0.1 M cacodylate buffer. Following a graded acetone dehydration series, the samples were embedded in Epon resin. Ultrathin sections were cut, stained with uranyl acetate and lead citrate, and examined using transmission electron microscopy (TEM; FEI Tecnai F20, Thermo, Hillsboro, OR, USA).

### 2.8. Scanning Electron Microscope

Duodenal mucosal morphology was examined using a cold-field emission scanning electron microscope (Regulus 8200, Hitachi, Tokyo, Japan). Briefly, tissue samples were rinsed twice in ice-cold PBS and fixed overnight at 4 °C in 2.5% glutaraldehyde. After fixation, specimens were trimmed into approximately 3 mm^3^ blocks, rinsed three times with chilled PBS, and then dehydrated through a graded ethanol series. The dehydrated samples were dried and sputter-coated with gold. Morphological observations and image acquisition were performed using the Hitachi SEM system.

### 2.9. 16S rRNA Sequencing and Data Processing

Cecal microbial DNA was extracted, and the V3–V4 region of the 16S rDNA libraries was constructed following Illumina’s standard metagenomic sequencing protocol, which amplifies the V3–V4 hypervariable region of the 16S rRNA gene. Sequencing was performed on an Illumina MiSeq platform to generate paired-end 250-bp reads. Data processing was conducted using Qiagen and the R environment. The raw sequencing was spliced and quality-controlled using FLASH v 1.20 and Trimmomatic v 0.36, with the following parameters: window size set to 50 bp, the average quality value of 20, and the minimum retained sequence length of 120 bp. The filtered data was merged with a minimum overlap of 10 bp, and the mismatch rate was 0.1, set via Pear v0.9.6. OTU clustering was performed in closed reference mode using the Vsearch v 2.7.1 package with QIIME2 v2023.9. Chao 1, Shannon, and ACE alpha diversity were computed using the OTU data. Furthermore, partial least squares discriminant analysis (PLS-DA) and non-metric multidimensional scaling (NMDS) were calculated at the OTU level using R software v4.5.0.

### 2.10. Statistical Analysis

Statistical analyses were performed using SPSS v 25.0 (IBM, New York, NY, USA). Data are presented as mean ± standard error (SE). In this study, the replicate cage was considered the experimental unit for growth performance data, whereas the individual sampled bird served as the experimental unit for physiological parameters and gut microbiota analyses. Differences among groups were assessed by one-way analysis of variance (ANOVA) followed by Duncan’s multiple comparison test. *p* < 0.05 was considered a significant difference. The correlation between gut microbiota and broilers’ physiological parameters, which include growth performance, antioxidative status, and lipid metabolism, was assessed using Spearman’s rank correlation analysis via OmicShare Tools (www.omicshare.com/tools, accessed on 31 July 2026).

## 3. Results

### 3.1. Effects of Dietary DNJ-Enriched Mulberry Extract on the Production Performance of Broilers Under a High-Fat Diet

**Figure 1:**
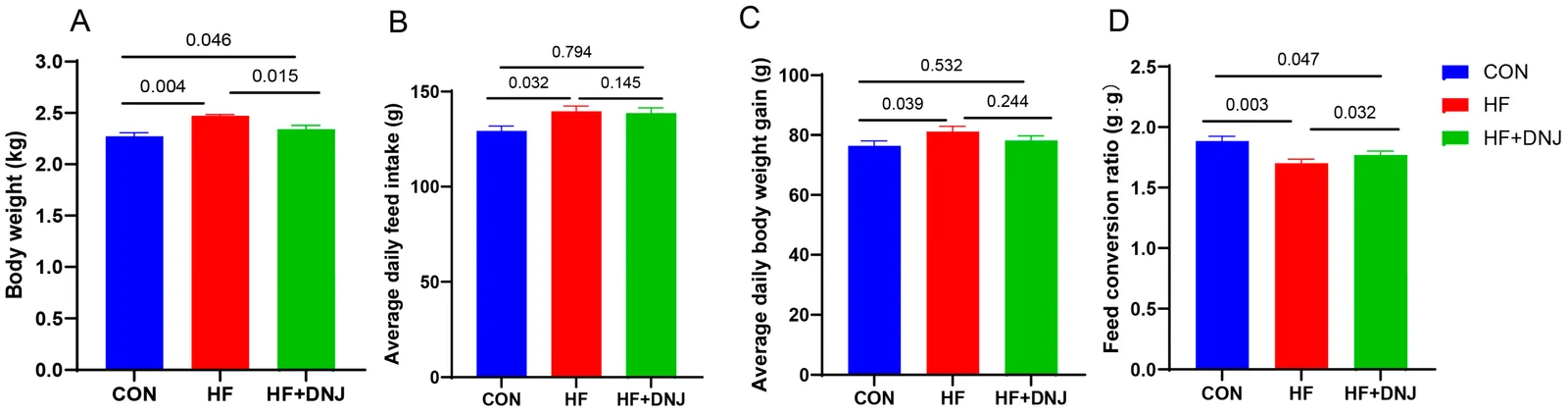
Effects of dietary DNJ-enriched mulberry extract on the growth performance of broilers fed a high-fat diet. (**A**) Body weight on day 42 (BW); (**B**) average daily feed intake (ADFI); (**C**) average daily body weight gain (BWG); and (**D**) feed conversion ratio (FCR). Data are presented as mean ± SEM. CON, control diet; HF, high-fat diet; HF + DNJ, high-fat diet supplemented with mulberry 1-deoxynojirimycin extract.

The production performance of broilers is illustrated in Figure 1. Compared to the CON group, the BW of broilers in the HF group increased significantly (*p* = 0.004); however, DNJ supplementation significantly reduced BW compared to the HF group (*p* = 0.015). HF treatment also significantly elevated ADFI and BWG (*p* = 0.032 and 0.039, respectively) compared to the CON group. DNJ supplementation numerically reduced ADFI and BWG compared with the HF group, although these differences were not statistically significant. Compared to the CON group, the HF group significantly decreased the FCR (*p* = 0.003). In contrast, DNJ supplementation significantly increased FCR compared with the HF group (*p* = 0.032), although FCR remained significantly lower than that in the CON group (*p* = 0.047).

### 3.2. Effects of Dietary DNJ-Enriched Mulberry Extract on the Liver Index and Antioxidant Parameters of Broilers Fed a High-Fat Diet

**Figure 2:**
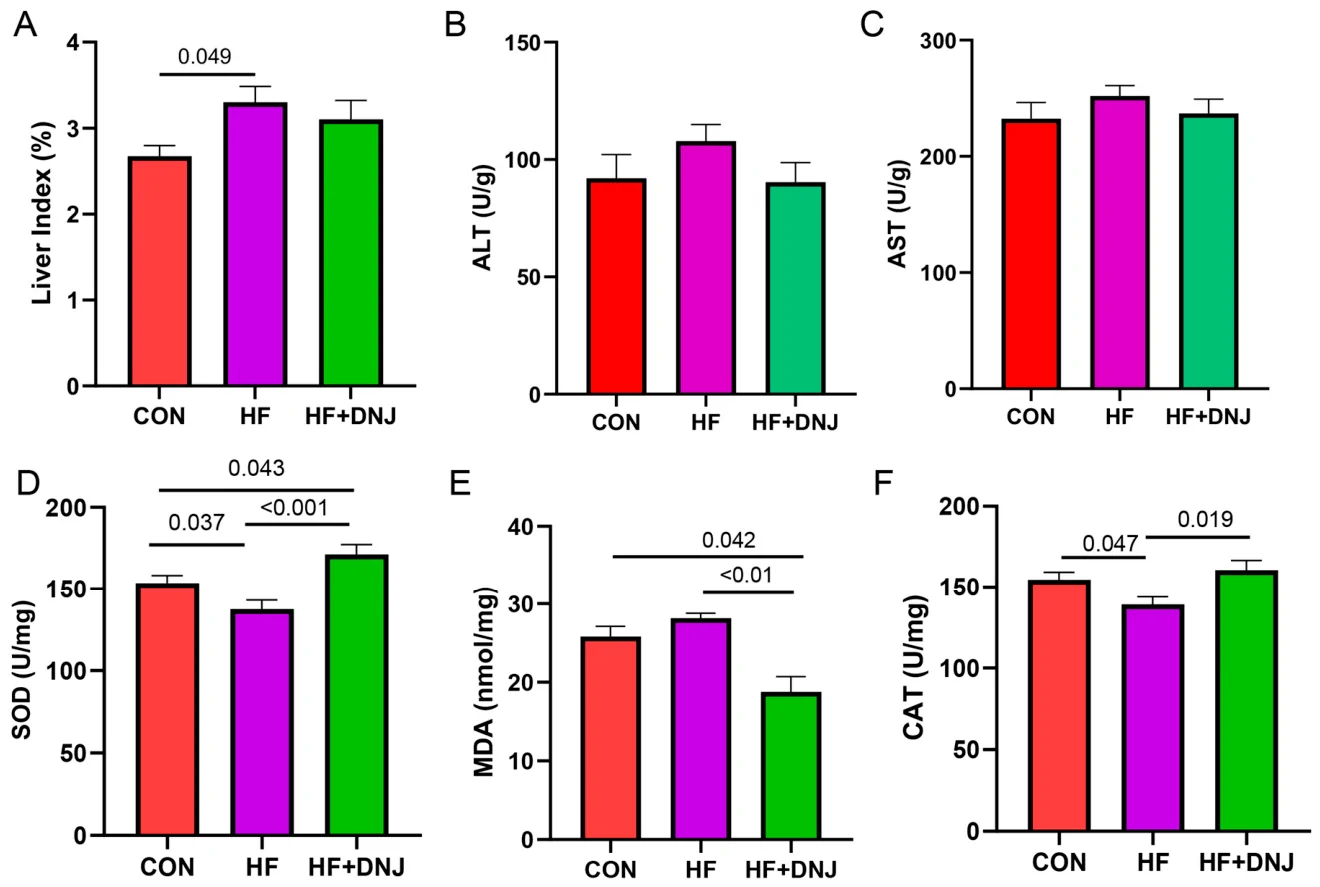
Effects of dietary DNJ-enriched mulberry extract on the liver index, hepatic enzyme activities, and antioxidant status of broilers fed a high-fat diet. (**A**) Liver index; (**B**) alanine aminotransferase (ALT) activity; (**C**) aspartate aminotransferase (AST) activity; (**D**) superoxide dismutase (SOD) activity; (**E**) malondialdehyde (MDA) content; and (**F**) catalase (CAT) activity. Data are presented as the mean ± SEM. CON, control diet; HF, high-fat diet; HF + DNJ, high-fat diet supplemented with mulberry 1-deoxynojirimycin extract.

As shown in Figure 2, the liver index was significantly increased in the HF group compared with the CON group (*p* = 0.049). In contrast, DNJ supplementation reduced the liver index compared with the HF group, although this reduction was not statistically significant. Regarding hepatic antioxidant status, HF treatment significantly reduced the levels of SOD and CAT (*p* = 0.037 and *p* = 0.047, respectively), while MDA content was not significantly affected. Conversely, DNJ supplementation significantly elevated SOD and CAT activities and decreased MDA content (*p* < 0.001, *p* = 0.019, *p* < 0.01, respectively).

### 3.3. Effects of Dietary DNJ-Enriched Mulberry Extract on Hepatic Lipid Metabolism and Histomorphology in Broilers Fed a High-Fat Diet

**Figure 3:**
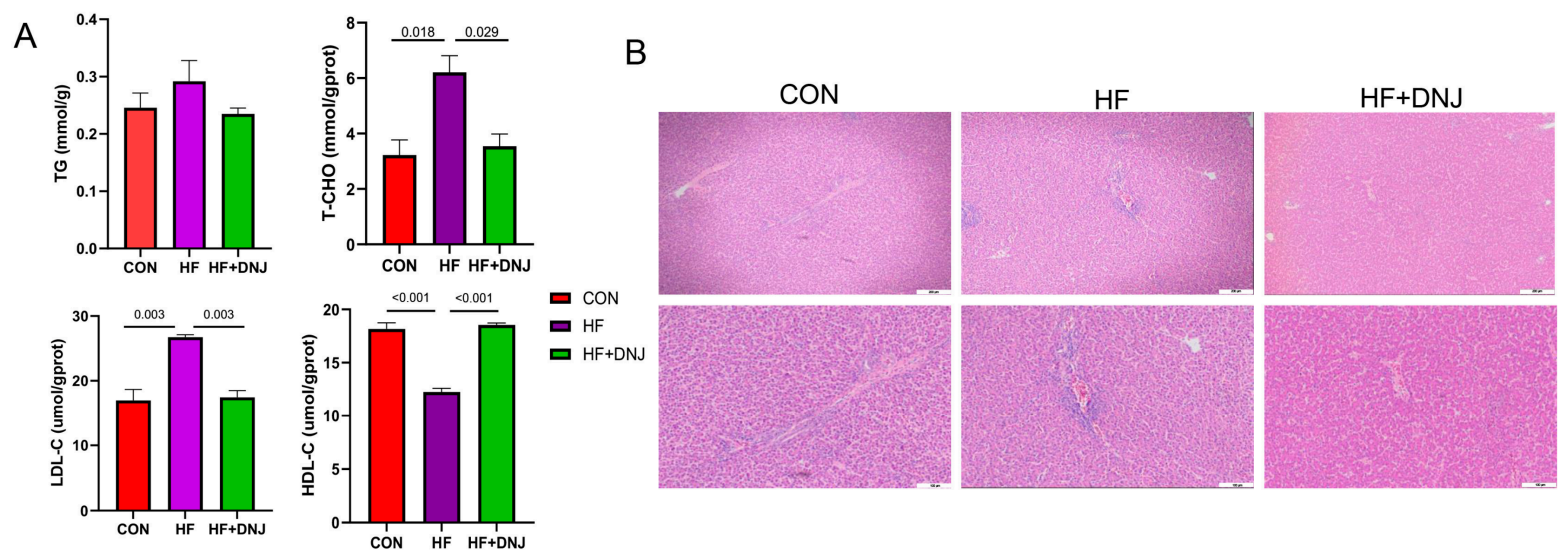
Effects of dietary DNJ-enriched mulberry extract on hepatic lipid metabolism and histomorphology in broilers fed a high-fat diet. (**A**) Hepatic levels of triglycerides (TG), total cholesterol (T-CHO), low-density lipoprotein cholesterol (LDL-C), and high-density lipoprotein cholesterol (HDL-C); (**B**) representative hematoxylin and eosin (H&E)-stained liver sections from the CON, HF, and HF + DNJ groups at low and high magnifications. Scale bars: upper row = 200 μm; lower row = 100 μm. Data are presented as the mean ± SEM. CON, control diet; HF, high-fat diet; HF + DNJ, high-fat diet supplemented with mulberry 1-deoxynojirimycin extract.

Lipid metabolism-related parameters are shown in Figure 3. Compared with the CON group, the HF group showed significantly increased hepatic T-CHO and LDL-C levels (*p* = 0.018 and 0.003, respectively) and a significantly decreased HDL-C level (*p* < 0.001). Compared with the HF group, DNJ supplementation significantly decreased T-CHO and LDL-C levels (*p* = 0.029 and *p* < 0.003, respectively) and increased HDL-C levels (*p* < 0.001).

Hepatic morphological observations in Figure 3 indicated that the liver sections from the CON group exhibited an intact hepatic architecture, with hepatocytes and hepatic sinusoids orderly arranged around the central veins and no apparent lipid vacuolation or inflammatory cell infiltration. In contrast, the HF group exhibited diffuse cytoplasmic vacuolation of hepatocytes, accompanied by hepatocellular enlargement, loosely arranged and lightly stained cytoplasm, and increased inflammatory cell infiltration in the portal areas and hepatic sinusoids. Compared with the HF group, the HF + DNJ group showed visibly reduced inflammatory cell infiltration and fewer cytoplasmic vacuoles.

### 3.4. Effects of Dietary DNJ-Enriched Mulberry Extract on the Intestinal Histomorphology of Broilers Fed a High-Energy Diet

As shown in Table 2, the HF diet significantly reduced duodenal crypt depth compared to the CON group, whereas duodenal villus height and the V/C ratio did not differ significantly among the groups (*p* = 0.346 and 0.190, respectively). DNJ supplementation further reduced duodenal crypt depth compared with the HF group, although this difference was not statistically significant based on the overall comparison.

In the jejunum, the HF diet significantly increased villus height and the V/C ratio compared with the CON group, whereas crypt depth was not significantly affected (*p* = 0.463). DNJ supplementation did not significantly alter jejunal villus height, crypt depth, or the V/C ratio compared with the HF group.

In the ileum, the HF diet significantly increased villus height, crypt depth, and the V/C ratio compared with the CON group. DNJ supplementation significantly reduced ileal crypt depth compared with the HF group (*p* = 0.019), whereas the differences in villus height and V/C ratio were not statistically significant.

### 3.5. Effects of Dietary DNJ-Enriched Mulberry Extract on the Ultrastructure of Intestinal Epithelial Cells in Broilers Fed a High-Fat Diet

**Figure 4:**
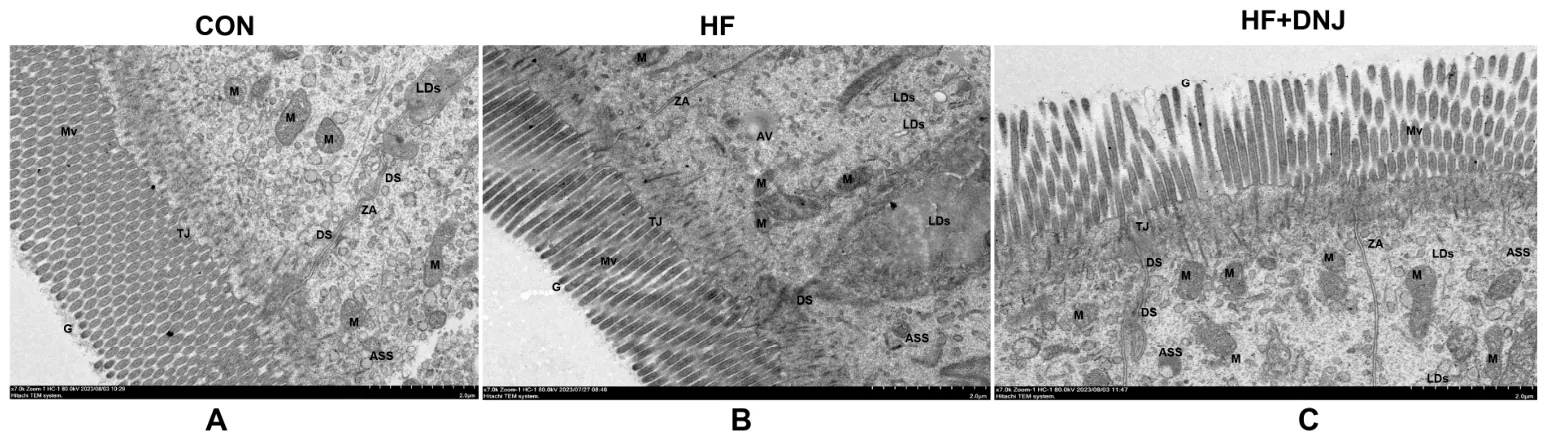
Effects of dietary DNJ-enriched mulberry extract on the ultrastructure of jejunal epithelial cells in broilers fed a high-fat diet. Representative transmission electron micrographs of jejunal epithelial cells from the (**A**) CON, (**B**) HF, and (**C**) HF + DNJ groups. Mv, microvilli; TJ, tight junction; ZA, zonula adherens; M, mitochondria; DS, desmosome; G, glycocalyx; LDs, lipid droplets; AV, autophagic vacuole; ASS, autolysosome. Scale bar = 2.0 μm. CON, control diet; HF, high-fat diet; HF + DNJ, high-fat diet supplemented with mulberry 1-deoxynojirimycin extract.

Transmission electron microscopy (TEM) observations are shown in Figure 4. Compared with the CON group, the HF diet induced cellular structural disorganization, including increased intracellular lipid droplet accumulation, abnormal organelle morphology, widened intermediate junctions, less distinct tight junction structures, and a reduced glycocalyx layer. Conversely, the HF + DNJ group showed an ultrastructural appearance more similar to that of the CON group, characterized by clearly defined tight junctions, narrowed intermediate junctions, fewer lipid droplet accumulations, and relatively preserved organelle morphology.

### 3.6. Effects of Dietary DNJ-Enriched Mulberry Extract on the Intestinal Surface of Broilers Fed a High-Fat Diet

**Figure 5:**
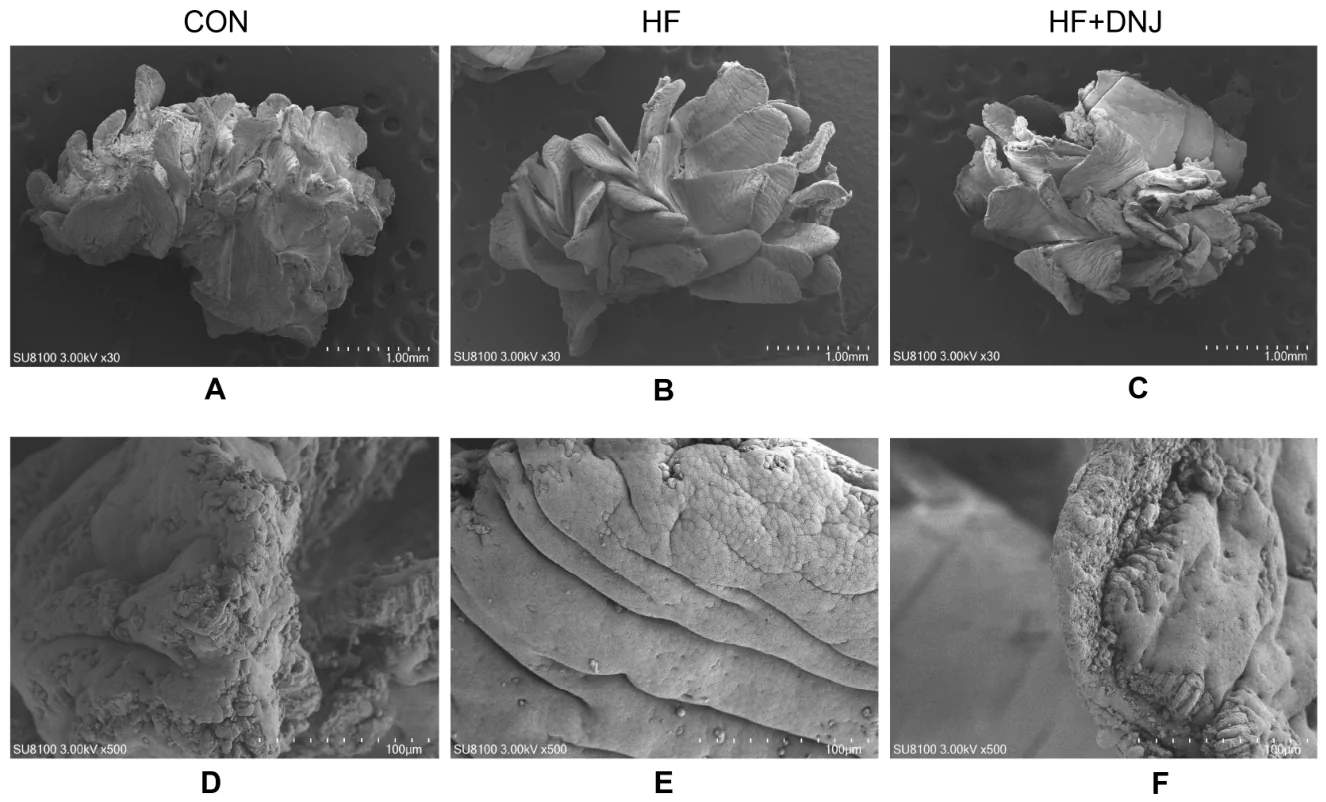
Effects of dietary DNJ-enriched mulberry extract on the duodenal mucosal surface of broilers fed a high-fat diet. Representative scanning electron micrographs of the duodenal mucosal surface. (**A**–**C**) Low-magnification images of the CON, HF, and HF + DNJ groups, respectively (×30; scale bar = 1.00 mm); (**D**–**F**) high-magnification images of the corresponding groups (×500; scale bar = 100 μm). CON, control diet; HF, high-fat diet; HF + DNJ, high-fat diet supplemented with mulberry 1-deoxynojirimycin extract.

Representative scanning electron microscopy (SEM) of the duodenal mucosal surface is shown in Figure 5. Compared with the CON group, the HF diet resulted in the foliaceous (leaf-like) intestinal villi broadening, while the goblet cells remained relatively aligned and smooth. In the HF + DNJ group, the villus morphology appeared more similar to that of the CON group, with a relatively narrower villus surface and a dense distribution of microvilli on the epithelial surface.

### 3.7. Effects of Dietary DNJ-Enriched Mulberry Extract on the Gut Microbiota of Broilers Fed a High-Fat Diet

**Figure 6:**
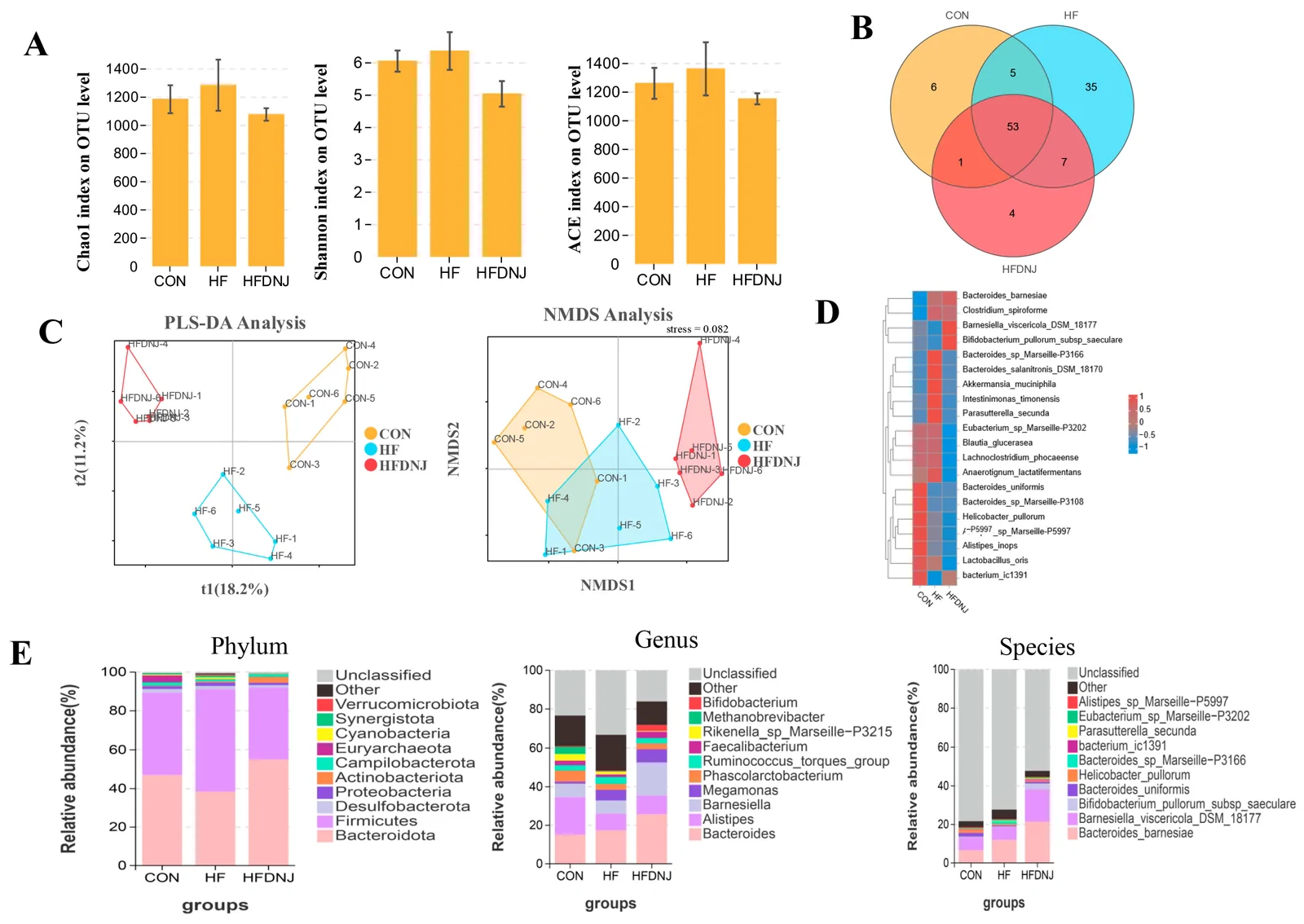
Effects of dietary DNJ-enriched mulberry extract on cecal microbial diversity and community composition in broilers fed a high-fat diet. (**A**) Alpha-diversity indices, including the Chao1, Shannon, and ACE indices; (**B**) Venn diagram showing the shared and unique operational taxonomic units (OTUs) among groups; (**C**) partial least-squares discriminant analysis (PLS-DA) and non-metric multidimensional scaling (NMDS) plots of microbial community structure; (**D**) clustered heatmap of the 20 most abundant bacterial species; and (**E**) relative abundances of the major bacterial taxa at the phylum, genus, and species levels. Data in panel (**A**) are presented as the mean ± SEM. CON, control diet; HF, high-fat diet; HF + DNJ, high-fat diet supplemented with mulberry 1-deoxynojirimycin extract.

To evaluate the impact of an HF diet on the intestinal microbiota diversity of broilers and to determine whether DNJ could mitigate these alterations, 16S rRNA sequencing was performed on cecal contents. As shown in Figure 6, the alpha diversity indices, including Chao1, Shannon, and ACE, were higher in the HF group and lower in the HF + DNJ group. However, no significant differences were observed among the groups (*p* > 0.05, Figure 6A). A total of 438 common operational taxonomic units (OTUs) were identified using a Venn diagram (Figure 6B), indicating that both HF and DNJ modulated the gut microbiota, resulting in substantial variations in community composition across groups. PLS-DA and NMDS analyses showed relatively tight clustering of samples within each group, whereas the distributions of samples among the three groups were visually separated (Figure 6C,D). Taxonomic profiling revealed differences in the relative abundances of several dominant bacterial taxa, including Bacteroidota, Firmicutes, *Bacteroides*, *Alistipes*, and *Bifidobacterium*-related taxa (Figure 6E).

**Figure 7:**
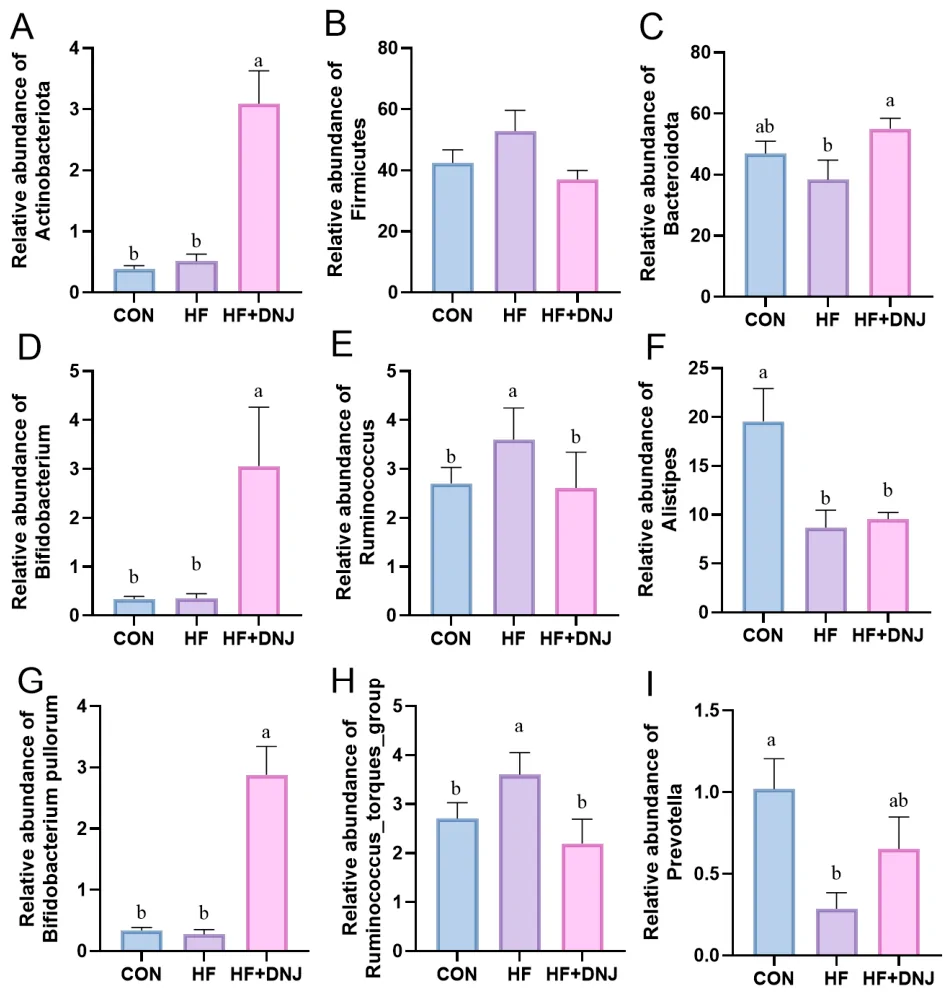
Effects of dietary DNJ-enriched mulberry extract on the relative abundances of selected cecal bacterial taxa in broilers fed a high-fat diet. Relative abundances of (**A**) Actinobacteriota, (**B**) Firmicutes, (**C**) Bacteroidota, (**D**) *Bifidobacterium*, (**E**) *Ruminococcus*, (**F**) *Alistipes*, (**G**) *Bifidobacterium pullorum*, (**H**) the *Ruminococcus torques* group, and (**I**) *Prevotella*. Data are presented as the mean ± SEM. Different lowercase letters indicate significant differences among groups (*p* < 0.05); bars without letters are not significantly different. CON, control diet; HF, high-fat diet; HF + DNJ, high-fat diet supplemented with mulberry 1-deoxynojirimycin extract.

To further identify specific taxonomic alterations among the treatments, the relative abundances of selected bacterial taxa were evaluated (Figure 7). At the phylum level, there was no significant difference in the relative abundance of Firmicutes among the three groups. Compared with the HF group, DNJ supplementation significantly increased the relative abundance of Bacteroidota (*p* < 0.05). Additionally, the relative abundance of Actinobacteriota was significantly enriched in the HF + DNJ group compared with both the CON and HF groups (*p* < 0.05). At the genus and species levels, the HF diet significantly elevated the relative abundances of *Ruminococcus* and the *Ruminococcus torques* group compared to the CON group (*p* < 0.05), whereas these abundances were significantly lower in the HF + DNJ group than in the CON group (*p* < 0.05). DNJ supplementation partially increased the relative abundance of *Prevotella*, although no significant difference was detected between the HF + DNJ group and either the CON or HF group (*p* > 0.05). In contrast, the relative abundance of *Alistipes* remained significantly lower in the HF + DNJ group than in the CON group (*p* < 0.05). Notably, the relative abundances of *Bifidobacterium* and *Bifidobacterium pullorum* were significantly higher in the HF + DNJ group than in both the CON and HF groups (*p* < 0.05).

### 3.8. Correlation Relationship Between Altered Microbiota and Growth Performance, Biochemical Indices, and Intestinal Morphology

**Figure 8:**
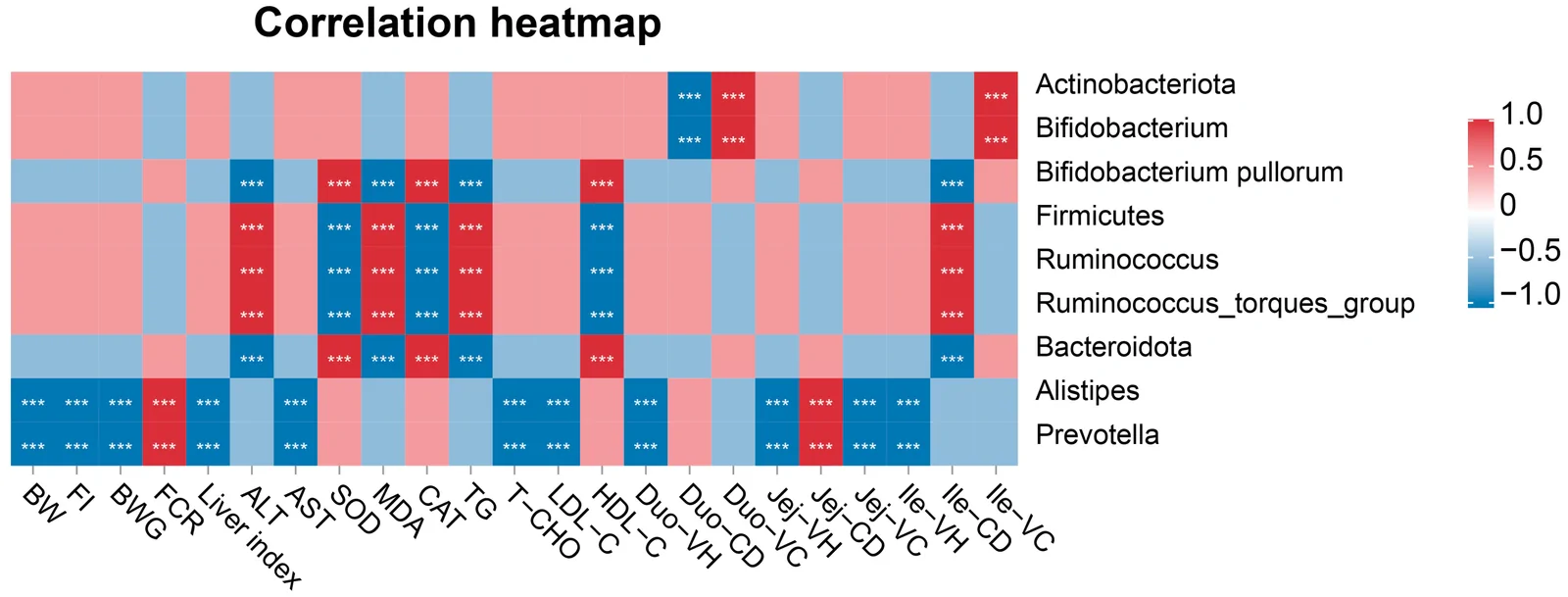
Spearman’s rank correlations between selected cecal bacterial taxa and the growth performance, hepatic biochemical indices, antioxidant status, lipid metabolism, and intestinal morphology in broilers. Red indicates a positive correlation, whereas blue indicates a negative correlation. BW, body weight; FI, feed intake; BWG, body weight gain; FCR, feed conversion ratio; ALT, alanine aminotransferase; AST, aspartate aminotransferase; SOD, superoxide dismutase; MDA, malondialdehyde; CAT, catalase; TG, triglycerides; T-CHO, total cholesterol; LDL-C, low-density lipoprotein cholesterol; HDL-C, high-density lipoprotein cholesterol; Duo, duodenum; Jej, jejunum; Ile, ileum; VH, villus height; CD, crypt depth; V/C, villus height-to-crypt depth ratio. *** *p* < 0.001.

To further elucidate the functional links between microbiota alterations and broiler physical parameters, a correlation heatmap was generated between key microbiota and these parameters (Figure 8). *Bifidobacterium pullorum* and Bacteroidota showed similar correlation patterns, with positive correlations with SOD, CAT, and HDL-C, and negative correlations with liver ALT, MDA, TG, and ileal crypt depth (Ile-CD). *Firmicutes*, *Ruminococcus*, and the *Ruminococcus torques* group were positively correlated (*p* < 0.001) with ALT, MDA, TG, and Ile-CD, and negatively correlated with SOD, CAT, and HDL-C (*p* < 0.001). Actinobacteriota and *Bifidobacterium* were significantly negatively correlated (*p* < 0.001) with T-CHO and LDL-C, while showing a significant positive correlation (*p* < 0.001) with ileal villus height (Ile-VH). Additionally, *Alistipes* and *Prevotella* were negatively correlated (*p* < 0.001) with growth performance and BW, FI, BWG, Liver index, AST, T-CHO, LDL-C, and intestinal villus height (Duo-VH, Jej-VH, Ile-VH), whereas positive correlations were observed with FCR (*p* < 0.001).

## 4. Discussion

The increasing use of high-energy diets in commercial poultry production has improved growth efficiency [23]; however, excessive dietary fat intake may impose metabolic challenges on broilers [2]. HF diets have been associated with disorders of lipid metabolism, oxidative imbalance, and impaired physiological functions in broilers [24]. In the present study, the HF diet was used as a nutritional challenge model to induce metabolic disturbances and evaluate the potential protective effects of DNJ-enriched mulberry extract. In practical production, identifying plant-derived nutritional strategies—such as epigallocatechin gallate [24] and echinocystic acid [25]—that can minimize the adverse effects of high-energy feeding remains an important objective in the poultry industry. DNJ is a naturally occurring alkaloid abundant in mulberry leaves and has been reported to exhibit multiple biological activities, including regulation of glucose and lipid metabolism and antioxidant activity [9]. Previous mammalian studies have shown that DNJ can regulate metabolic disorders associated with excessive nutrient intake [26,27], supporting its potential application in animal nutrition. In the present study, DNJ supplementation partially counteracted several HF diet-associated alterations in growth performance, hepatic lipid metabolism, antioxidant status, intestinal morphology, and gut microbial composition, suggesting that DNJ may provide multifaceted metabolic benefits under a high-fat nutritional challenge.

It should be noted that the FCR observed in this study was higher than the standard performance objectives for commercial AA broilers. This discrepancy may be primarily attributed to the use of mash diets, which were necessary to ensure the homogeneous mixing of the experimental substances [28]. Nevertheless, all groups were subjected to identical housing and feeding conditions, allowing the relative responses among dietary treatments to be evaluated under comparable experimental conditions. Additionally, regarding production performance, evaluating processing yield data would provide valuable insights into how DNJ modulates energy partitioning under high-fat conditions. The present experiment primarily focused on the effects of DNJ on internal metabolic and intestinal physiological responses, rather than carcass characteristics. Given that excessive dietary lipid intake may disturb hepatic lipid homeostasis and impair intestinal integrity, the potential effects of DNJ on these two major metabolic organs were subsequently investigated.

The liver plays a central role in lipid synthesis, oxidation, and transport, making it particularly susceptible to excessive dietary lipid intake. In poultry, excess lipid availability promotes fatty acid uptake and triglyceride synthesis in the liver [25,29]. Consistent with this metabolic response, the HF diets in the present study altered lipid-related parameters, as reflected by increased T-CHO and LDL-C levels and reduced HDL-C levels. In contrast, DNJ supplementation decreased these indicators and improved hepatic morphology, aligning with previous studies in mice [25,26]. However, because hepatic lipid deposition and lipid synthesis pathways were not directly quantified in the present study, the precise metabolic processes responsible for these changes remain to be determined. Disorders of lipid metabolism are frequently accompanied by increased reactive oxygen species (ROS) production and lipid peroxidation, which can damage cellular structures and physiological processes, and increased MDA levels [30]. Antioxidant enzymes, including SOD and CAT, constitute an important defense system against excessive ROS accumulation [31]. In the present study, the HF diet decreased hepatic SOD and CAT activities, whereas DNJ supplementation increased both enzyme activities and reduced MDA content. These findings indicate that DNJ supplementation was associated with an improved hepatic antioxidant status under HF dietary conditions. However, whether DNJ directly activates endogenous antioxidant signaling or indirectly improves redox homeostasis through the regulation of lipid metabolism requires further investigation.

Maintaining intestinal barrier function is essential for preserving animal health; excessive dietary lipid intake may impair intestinal integrity through oxidative stress, epithelial disruption, and alterations in tight junction structure [32]. Our previous studies have demonstrated that DNJ exerts beneficial effects on intestinal morphology and ultrastructure under oxidative stress induced by H_2_O_2_ [15]. In this study, we found that HF diet feeding negatively affects intestinal morphology. In contrast, DNJ supplementation improved villus structure and restored intestinal barrier characteristics, further confirming the protective role of DNJ in the intestine. Villi influence the digestive and absorptive functions of the intestine and maintain intestinal integrity and immune function [33]. Few studies have focused on changes in intestinal villus morphology, with most concentrating on villus length and ultrastructure. Our results indicate that DNJ supplementation improves the intestinal surface, particularly villus morphology. These findings clearly demonstrate that DNJ affects various aspects of intestinal function.

Gut function is not only related to barrier integrity but also to the balance of the gut microbiota. A study in geese found that 150 mg/kg of mulberry leaf DNJ increased the relative abundance of *Bacteroides* and reduced that of *Ruminococcaceae*, but impaired growth performance [16]. Our study found that adding 80 mg/kg increased the abundance of Actinobacteriota and Bacteroidota. Bacteroidota are key producers of short-chain fatty acids (SCFAs) and play a regulatory role in improving gut microbiota, which has previously been associated with intestinal health and metabolic regulation [34]. A higher Firmicutes/Bacteroidota ratio indicates a certain degree of intestinal barrier damage [35]; in our study, the Firmicutes/Bacteroidota ratio decreased significantly after DNJ supplementation (calculated according to data in Figure 7), a result similar to that observed in high-fat-fed mice [36]. Further analysis revealed that the abundance of *Bifidobacterium* and its chicken-specific species, *Bifidobacterium pullorum*, both increased significantly. *Bifidobacterium* has been recognized as a beneficial microorganism due to its contribution to intestinal barrier maintenance and immune regulation [37]. Due to the limited number of studies on specific microbiota colonization, the functions of these microorganisms are not yet thoroughly understood, warranting further investigation.

The gut microbiome influences human health through the gut-liver axis by producing various metabolites resulting from dynamic changes in the microbial community [38]. Consequently, the gut-liver relationship has become a prominent research focus in recent years [39]. Our correlation analysis results also indicate that Bacteroidota is significantly positively correlated with the antioxidant markers of SOD, CAT, and the good cholesterol of HDL-C, while *Firmicutes* is negatively correlated with these markers and positively correlated with markers such as MDA, ALT, and TG. These findings suggest that DNJ can reduce *Firmicutes* abundance by increasing Bacteroidota abundance, thereby regulating oxidative stress and lipid metabolism disorders induced by a high-fat diet. Overall, this study demonstrates that DNJ-enriched mulberry extract alleviates HF diet-induced oxidative stress in broilers by improving hepatic lipid metabolism, antioxidant capacity, intestinal barrier integrity, and gut microbial composition. The role exerted may involve the interaction between the liver and the intestine. However, the detailed underlying mechanisms require further investigation, particularly focusing on microbial metabolites and specific metabolic pathways that regulate the gut-liver axis, to better define the practical application potential of DNJ in poultry health. Furthermore, it is worth noting that this study focused primarily on the role of DNJ under HF diet-induced stress. Although previous studies have confirmed the safety of DNJ in poultry, the absence of a normal diet plus DNJ group limits the evaluation of its basal metabolic effects in healthy broilers, which should be addressed in future comprehensive toxicological evaluations.

## 5. Conclusions

In conclusion, dietary supplementation with mulberry-derived DNJ extract mitigated liver damage and intestinal injury in broilers fed a high-fat diet. DNJ supplementation reduced excessive lipid deposition and MDA levels while increasing SOD and CAT activities in the liver. Additionally, DNJ supplementation improved mucosal morphology and maintained epithelial barrier function. Alterations in cecal microbial composition further indicated that DNJ regulates microbiota abundance and interactions within the liver–gut axis. Overall, this study highlights the potential value of DNJ-enriched mulberry extract as a plant-derived substance to promote liver and intestinal health in broilers exposed to nutritional stress.

## Figures and Tables

**Table 1:** Ingredient composition and nutrient levels of starter diets, as well as grower-stage diets with a high-fat diet (air-dried basis).

Ingredient	1–21 d	22–42 d	
		Con	HF
Corn	59	60.3	58.8
Soybean Meal (CP 44%)	35	33	31.9
Soybean oil	1.8	3	5.6
Calcium carbonate	1.48	1.3	1.3
Calcium hydrophosphate	1.66	1.32	1.32
L-Lys-HCL (98%)	0.16	0.18	0.18
DL-Met	0.2	0.2	0.2
Salt	0.3	0.3	0.3
Premix Vitamin ^1^	0.1	0.1	0.1
Premix Mineral ^2^	0.3	0.3	0.3
Total	100	100	100
Level of nutrients (calculated)	1–21 d	22–42 d	
ME (kcal/kg)	2980	3110	3240
CP (%)	21.06	19.66	19.68
EE (%)	3.45	6.25	8.29
Ca (%)	1.00	0.95	0.95
Av. P(%)	0.46	0.39	0.39
Lys (%)	1.21	1.06	1.06
Met (%)	0.53	0.42	0.43
Met + cys (%)	0.85	0.76	0.75

^1^ Per kg of premix provides: VA 12,000 IU/kg; VD 33,000 IU/kg; VE 7.5 IU/kg; VK 31.50 mg/kg; VB1 0.6 mg/kg; VB2 4.8 mg/kg; VB6 1.8 mg/kg; VB12 10 mg/kg; Folic acid 0.15 mg/kg; niacinamide 30 mg/kg; pantothenic acid 10.5 mg/kg. ^2^ Fe 80 mg, Cu 8 mg, Mn 80 mg, Zn 60 mg, Se 0.15 mg, I 0.35 mg. Abbreviations: ME, Metabolizable Energy; CP, Crude Protein; EE, Ether Extract; Ca, Calcium; Av. P, Available Phosphorus; Lys, Lysine; Met, Methionine.

**Table 2:** Effects of DNJ-enriched mulberry extract on the intestinal morphology in broilers fed a high-fat diet.

Organ	Item	Control	HF	HF + DNJ	S.E.M	*p*-Value
Duodenum	Villus height (μm)	1450.33	1553.25	1538.5	32.13	0.346
	Crypt dept (μm)	109.17 ^a^	84.625 ^b^	77.25 ^b^	4.87	0.009
	V/C	14.92	18.41	26.30	2.60	0.19
Jejunum	Villus height (μm)	1250.27 ^b^	1562.63 ^a^	1471.25 ^a^	32.87	<0.001
	Crypt dept (μm)	82.36	75.86	77.50	22.5	0.463
	V/C	15.61 ^b^	21.24 ^a^	19.22 ^a^	0.811	0.007
Ileum	Villus height (μm)	879.67 ^b^	1342.83 ^a^	1265.33 ^a^	55.70	<0.001
	Crypt dept (μm)	78.67 ^a^	81.11 ^a^	66.67 ^b^	2.22	0.019
	V/C	11.31 ^b^	16.66 ^a^	19.06 ^a^	0.80	<0.001

Note: V/C, ratio of villus height/crypt dept. Different letters represent a significant difference within groups.

## Data Availability

All data generated or analyzed during this study are included in this paper and are available from the corresponding authors upon reasonable request.

## Supplementary Materials

The following supporting information can be downloaded at: https://www.mdpi.com/article/10.3390/ani16182872/s1, Figure S1. HPLC profile for DNJ-enriched mulberry extract.
